# Pathogenic, Phylogenetic, and Serological Analysis of Group I Fowl Adenovirus Serotype 4 SDSX Isolated From Shandong, China

**DOI:** 10.3389/fmicb.2018.02772

**Published:** 2018-11-19

**Authors:** Guanliu Yu, Yawen Wang, Minmin Zhang, Yun Lin, Yi Tang, Youxiang Diao

**Affiliations:** ^1^College of Animal Science and Technology, Shandong Agricultural University, Tai'an, China; ^2^Shandong Provincial Key Laboratory of Animal Biotechnology and Disease Control and Prevention, Shandong Agricultural University, Tai'an, China; ^3^Shandong Provincial Engineering Technology Research Center of Animal Disease Control and Prevention, Shandong Agricultural University, Tai'an, China

**Keywords:** FAdV-4, hydropericardium hepatitis syndrome, meat duck, pathogenicity, real-time polymerase chain reaction, virus infection

## Abstract

Hydropericardium hepatitis syndrome (HHS) caused by group I fowl adenovirus serotype 4 (FAdV-4) is an acute and infectious disease in fowl, particularly in broilers aged 3–5 weeks. In June 2015, a highly pathogenic disease outbroke in 25–40 day-old ducklings in Shandong province, characterized similar symptom to HHS. In order to determine the pathogenic mechanism of FAdV-4 (SDSX strain) in meat ducks. We divided 90 25-day-old Cherry Valley meat ducks into three groups (oral, subcutaneous, and control; 30 ducks in each group) and infected them with the virus. HHS, inclusion body hepatitis, and enlargement and hemorrhage of the spleen, kidney, lung, thymus, and brain were observed in FAdV-4-infected meat ducks. Histopathological changes were mainly characterized by severe fatty degeneration in the liver, basophilic inclusion bodies in hepatocytes, and vacuolation in the bursa. More importantly, viral DNA could be detected by quantitative real-time polymerase chain reaction in several viscera tissues (e.g., heart, liver, spleen) on the third day after infection. Notably, the livers of the two infected groups contained the highest concentration of viral DNA. In addition, immune responses were studied based on titer levels of the virus antibody and the levels of inflammatory cytokines interleukin (IL)-2 and interferon (IFN)-γ, and most levels were significantly upregulated, indicating that the host immune responses were activated early in infection. These findings increase our understanding of the pathogenicity of FAdV-4 (SDSX) in meat ducks and provide the foundation for further in-depth study of the pathogenic mechanism of this virus.

## Introduction

Fowl adenovirus serotype-4 (FAdV-4) is a member of the *Aviadenovirus* genus, *Adenoviridae* family, and contains non-enveloped and double-stranded DNA with a genome of approximately 43–46 kb that encodes 11 non-structural proteins [E1A, E1B, E2A (DBP), E3 (ADP), E4, EP, 33 K, 52/55 K, pol, pIVaII, and 100 K] and 10 primary structural proteins (hexon, penton base, fiber, terminal protein, protein μ, protein IIIa, protein V, protein VI, protein VII, and protein VIII; Griffin and Nagy, 2011; Xie et al., 2013; Li et al., 2017). This virus can cause acute avian infectious diseases such as hydropericardium hepatitis syndrome (HHS), inclusion body hepatitis (IBH), and gizzard erosion in duck layers and broilers (Marek et al., 2012; Chen et al., 2016). Among these diseases, HHS was first reported in Angara Goth (also named Angara disease), Pakistan in 1987 and subsequently broke out in North America, Mexico, Eastern Europe, India, South Asia, China, Japan, and South Korea, causing economic losses mainly to the broiler industry worldwide (Al-Sadi et al., 2000; Kim et al., 2008; Mase et al., 2009; Kaján et al., 2013; Niczyporuk, 2016).

Since 2015, severe outbreaks of HHS have occurred in commercial duck farms in Jiangsu, Anhui, Hubei, Jiangxi, Shandong, and Henan Provinces, China, resulting in high mortalities, pericardial effusion, and stunted growth among ducks (Chen et al., 2016; Yu et al., 2018). Several previous studies have focused on the epidemiology (Liu et al., 2015; Zhang et al., 2016; Li et al., 2017), gene structure (Mase et al., 2009; Griffin and Nagy, 2011; Shah et al., 2016), diagnostic detection (Günes et al., 2012; Junnu et al., 2014; Niczyporuk et al., 2015; Wang et al., 2017), and vaccine strategies (Schonewille et al., 2010; Shah et al., 2012, 2017) for the disease. Unfortunately, little is known about the pathogenicity of this virus in Cherry Valley meat ducks, except that intracerebral infection caused a 15% mortality rate (Chen et al., 2016) and oral infection showed no clinical signs (Pan et al., 2017); therefore, to complete the pathogenicity of the virus, Cherry Valley meat ducks were infected with FAdV-4 in this study. Ducks aged 25 days were infected with the virus both orally and by subcutaneous injection. The clinical symptoms, weight changes, pathological changes, viral distribution, serum biochemical indicators, and phylogenetics of hexon were then systematically analyzed. This study laid the foundation for further in-depth study of the pathogenic mechanism of FAdV-4 and contributed to the control of this contagious disease.

## Materials and methods

### Virus

Duck-origin FAdV-4 [SDSX, National Center for Biotechnology Information (NCBI) GenBank Accession No. KT899325] was isolated from dead ducks in 2015 in Shenxian County, Shandong Province, China, and stored. The median embryo infectious dose (EID_50_) of the SDSX strain was 10^−7.569^/0.2 ml, which was calculated using the Reed–Muench assay (Reed and Muench, 1938).

### Experimental design

In this study, 90 healthy Cherry Valley ducks (20 day old) were purchased from the Liuhe poultry hatchery (Shandong, China) and were reared for 5 days to ensure normal growing status before inoculation. Cloacal/tracheal swabs and serum samples were collected from ducks and tested by polymerase chain reaction (PCR) to confirm that they were free of FAdV-4 infection. In addition, other duck pathogens (i.e., avian reovirus virus, avian influenza virus, duck tembusu virus, duck plague virus, duck hepatitis A virus type-1, muscovy duck parovirus, and newcastle disease virus) tested negative by PCR; this guaranteed that our experimental ducks did not have any other infection.

The Cherry Valley ducks were randomly divided into three groups (oral, subcutaneous, and control), with 30 ducks in each group. Each duck was inoculated with 1 mL FAdV-4 (SDSX strain; ELD_50_, 10^−7.569^/0.2 ml) either orally or by neck subcutaneous injection. The control group was inoculated with equal doses of phosphate buffered saline (PBS) at the same injection site. The three groups were separately reared in different animal houses and on a net placed 0.5 m above the ground. Water and food were autoclaved before feeding and automatically refilled. The temperature of each house was maintained using radiators at 20–24°C. The houses were naturally ventilated, and the duck feces were manually cleaned.

All the animal experiments were performed according to the guidelines of the Committee on Ethics of Animals of Shandong and the appropriate biosecurity, and the Animal Care and Use Committee of Shandong Agricultural University approved the protocol (No. SDAUA-2016-208).

### Body weight and serum biochemical indicators

The body weights of 10 ducks from each group were measured using an electronic scale at 3, 6, 9, 12, and 15 days post-infection (dpi).

Blood (3 ml) was collected from each duck from the medial metatarsal vein at 3, 6, 9, 12, and 15 dpi (8 ducks in each group) and put into ethylenediaminetetraacetic acid (EDTA) vacuum tubes. After centrifuging for 15 min at 1,000 g, the serum samples were stored at −20°C until analysis.

Duck interleukin (IL)-2 and interferon (IFN)-γ detection kits purchased from Langton Biological Technology Co., Ltd. (Shanghai, China) were used to detect the IL-2 and IFN-γ levels in the blood samples. The levels of alkaline phosphatase (ALP), alanine transaminase (ALT), aspartate transaminase (AST), lactate dehydrogenase (LDH), and urea were detected by Adicon Clinical Laboratories, Inc. (Jinan, China). Each sample was analyzed in triplicate.

### FAdV-4 antibody

FAdV-4 antibody levels in the serum were detected by enzyme-linked immunosorbent assay (ELISA). In the assay, FAdV-4 recombinant hexon protein was employed as antigen to detect antibodies to SDSX strain according to the manufacturer's recommended instructions. Initially, the optimal dilution of the antigen and serum were determined by a checker board titration with duck FAdV-4-positive and -negative sera. The purified hexon protein diluted in 0.05 M carbonate buffer (pH 9.6) were coated separately in 96-well plates ranging from 0.2 to 200 ng/μl. The dilutions of duck serum samples were ranged from 1:25 to 1:800. Both reference positive and negative sera were diluted serially 2-fold and tested in separate plates. Dilutions that resulted in the maximum difference in absorbance at 450 nm for the positive and negative sera (P/N) were defined as the optimal working conditions to test the experimental serum samples.

Briefly, 100 μl purified hexon protein (50 ng/μl, prepared in our previous study) put into each well of the ELISA plates (Greiner Bio-One, Germany) and incubated for 12 h at 4°C. Then, the plates were washed three times with washing buffer (PBS containing 0.05% Tween-20, PBST) and shaken on a rocking bed, after which they were blocked with 200 μL/well 5% skim milk powder (Solarbio, Beijing, China; w/v) and incubated for 2 h at 37°C. After blocking, the plates were washed three times with PBS and shaken again using a rocking bed, after which duck serum (1:10) was added into the wells at 100 μL/well and incubated for 1 h at 37°C. The plates were washed again three times with PBS, incubated with rabbit anti-duck immunoglobulin G (IgG) prepared by our lab at 1:1000, and conjugated with horseradish peroxidase (Sigma-Aldrich Corporation, St. Louis, MO, USA) at 100 μl/well. The plates were incubated again for 1 h at 37°C and were washed three times with PBS, after which 100 μl 3′3′5′5′-tetramethylbenzidine (TMB) substrate solution (TransGen, Beijing, China) was added to each well and left to sit for 25 min in the dark. Stop buffer (50 μl; 3 M H_2_SO_4_) was added to stop the reaction, and the optical density (OD) values were measured at 450 nm using an automated ELISA plate reader (Thermo Fisher Scientific, Waltham, MA, USA). The cutoff value was an OD of 0.402 at 450 nm. OD values of the positive control (OD_pos_) and the samples (OD_sample_) were corrected by subtracting the OD value of the negative control (OD_neg_). Sample value was calculated as a ratio using the formula: value = (OD_sample_-OD_neg_)/(OD_pos_-OD_neg_). Each sample was analyzed in triplicate.

### Genomic DNA extraction and PCR

The total viral DNA was extracted from the visceral tissue samples using a DNeasy Tissue kit (Qiagen, Hilden, Germany), according to the manufacturer's instructions. A DeNovix DS-11 Spectrophotometer (Nanodrop, USA) was used to detect the concentration and quality of the extracted DNA, which was stored at −20°C until use.

The FAdV-4 genotype was detected according to the procedures outlined in our previous study (Tang et al., 2009). The entire hexon open reading frame of the SDSX strain was amplified by PCR using *TransScript*^®^ DNA Polymerase High Fidelity (HiFi; TransGen Biotech, Beijing, China) with two pairs of primers (Table 1), which were designed following conserved sequences of the L1 region of the hexon gene in the NCBI GenBank database.

**Table 1:** Primers and probe used in this study.

**Primer**	**Sequence (5′-3′)**	**Size (bp)**	**Purpose**
Hexon	F1: TGGACATGGGGGCGACCTA	1219	Hexon gene amplification
	R1: AAGGGATTGACGTTGTCCA		
	F2: AACGTCAATCCCTTCAACCACC	1350	
	R2: TTGCCTGTGGCGAAAGGCG		
FAdV-4	F: CGTCAACTTCAAGTACTC	86	TaqMan-based real-time PCR
	R: AGAGGATGCTCATGTTAC		
	Probe: FAM-CCTACTCAGATGGAGGCTTCTACC-TAMRA		

PCR was conducted in a reaction volume of 50 μl containing 3 μl viral DNA, 2 μl of dNTP (0.2 mmol/L), and MgCl_2_ (8 mmol/L), and each primer (100 mmol/L) contained 5 μL 10 × PCR buffer and 0.25 μl Ex *Taq* DNA polymerase (Takara, Dalian, China). The PCR conditions were as follows: initial incubation for 5 min at 94°C, followed by 33 cycles for 50 s at 94°C, anneal for 60 s at 56°C, and extension for 120 s at 72°C, and then an extension for 8 min at 72°C. PCR products were visualized by electrophoresis in a 0.9% (w/v) agarose gel containing ethidium bromide and subsequently purified and cloned into pEASY-T1 vector (TaKaRa, Dalian, China), according to the manufacturer's instructions. Then, the positive clone was sequenced (Sangon Biotech, Shanghai, China).

### Quantitative real-time PCR (qPCR) for virus DNA concentration

FAdV-4 viral DNA concentration was measured from visceral tissues (i.e., heart, liver, spleen, lung, kidney, bursa, thymus, pancreas, intestine, and brain) in ducks infected with FAdV-4 (SDSX) at 3, 6, 9, 12, and 15 dpi using a TaqMan-based real-time PCR with the 7,500 Fast Real-Time PCR System (Applied Biosystems, CA, USA). The primers and probe were designed following the conserved sequences of FAdV-4-specific L1 region of the hexon gene in the NCBI GenBank database (Table 1) and were synthesized by Sangon Biotech (Shanghai, China). To confirm the number of FAdV-4 copies in the visceral tissues of the infected ducks, viral DNA concentration (log_10_) was normalized as FAdV-4 copies of total DNA per μg. Furthermore, real-time PCR was performed using the *Premix Ex Taq*^TM^ kit (TaKaRa, Dalian, China) in a volume of 25 μl, following the manufacturer's instructions. The amplification program was set as follows: stage 1, 95°C for 30 s; stage 2, 40 cycles of denaturation at 95°C for 5 s and 60°C for 34 s. Each sample was analyzed in triplicate.

### Histopathology observation

At 12 dpi, 14 infected ducks (seven in each group) and seven control ducks were euthanized. Visceral tissue samples (e.g., heart, liver, spleen, lung, kidney, and thymus) from the different groups were collected into 10% neural buffered formalin and were cut into 4- to 5-mm-thick sections, stained with hematoxylin and eosin, and embedded in paraffin using standard methods. Finally, histopathological changes were observed under a light microscope.

### Phylogenetic analyses

The ClustalW method in the MegAlign program of DNAStar was used to align the amplified fragments of hexon. Phylogenetic trees were created based on the complete FAdV-4 hexon gene, which included the SDSX strain and 16 other reference strains (NCBI GenBank; Table 2, **Figure 8**), through neighbor-joining analysis using MEGA 5.05, where bootstrap confidence values were 1,000 replicates (Tamura et al., 2011).

**Table 2:** Sequence identity among viruses isolated in this study and with previously identified virus isolates.

		**%Nucleotide identity**																
	**% Amino acid identity**	**1**	**2**	**3**	**4**	**5**	**6**	**7**	**8**	**9**	**10**	**11**	**12**	**13**	**14**	**15**	**16**	**17**
1	KX247012 (61/11Z)	100	99.7	99.7	74.3	74.3	75.9	75.9	75.9	75.9	76.0	73.0	73.0	76.3	76.6	76.1	78.5	54.6
2	KX247011 (W-15)	99.7	100	99.9	74.2	74.2	75.8	75.9	75.8	75.8	75.9	72.9	72.9	76.3	76.5	76.0	78.5	54.5
3	U46933	99.8	99.9	100	74.2	74.2	75.9	75.9	75.8	75.9	75.9	72.9	72.9	76.3	76.5	76.0	78.5	54.5
4	NC021221 (340)	80.9	80.9	81.0	100	100.0	72.8	73.0	72.8	72.8	72.9	76.7	76.7	78.3	78.4	78.1	75.6	54.7
5	KC493646 (340)	80.9	80.9	81.0	100	100	72.8	73.0	72.8	72.8	72.9	76.7	76.7	78.3	78.4	78.1	75.6	54.7
6	U26221	76.9	76.9	76.9	73.5	73.5	100	97.5	97.3	97.1	97.2	72.0	72.0	74.9	75.1	74.8	78.3	55.4
7	HE608152 (KR5)	81.2	81.0	81.0	77.6	77.6	92.0	100	99.0	98.8	98.9	71.8	71.8	74.9	75.0	74.7	78.7	55.3
8	EU931693 (PK-01)	81.5	81.4	81.4	77.4	77.4	91.7	98.9	100	99.8	99.8	71.9	71.9	74.7	74.8	74.7	78.7	55.2
9	KT899324 (SDJX)	81.4	81.3	81.3	77.2	77.2	91.3	98.5	99.6	100	99.9	71.8	71.8	74.8	74.9	74.7	78.7	55.2
10	KT899325 (SDSX)	81.5	81.4	81.4	77.4	77.4	91.4	98.7	99.8	99.8	100	71.9	71.9	74.8	74.9	74.8	78.8	55.2
11	KU746335 (MX95-S11)	80.7	80.6	80.6	87.4	87.4	73.5	77.6	77.7	77.6	77.7	100	100	78.6	78.7	78.5	72.8	53.9
12	KM096545 (HBQ12)	80.7	80.6	80.6	87.4	87.4	73.5	77.6	77.7	77.6	77.7	100	100	78.6	78.7	78.5	72.8	53.9
13	KX077988 (HLJ/151129)	82.2	82.1	82.1	86.6	86.6	74.5	78.6	78.4	78.3	78.4	90.5	90.5	100	99.2	97.4	77.1	55.4
14	GU734104 (HG)	82.1	82.0	82.0	86.6	86.6	74.4	78.5	78.4	78.3	78.4	90.4	90.4	99.6	100	97.5	77.2	55.4
15	KX258422 (FV211-16)	82.2	82.1	82.1	86.3	86.3	74.4	78.3	78.4	78.3	78.4	90.4	90.4	98.2	98.3	100	77.0	53.3
16	GU936707 (D90/2)	82.9	82.9	82.9	89.0	79.0	75.5	79.2	79.4	79.2	79.3	79.7	79.7	80.2	80.1	80.1	100	55.9
17	NC001405	49.3	49.3	49.3	47.8	47.8	47.2	48.2	48.4	48.4	48.5	47.9	47.9	48.2	48.2	48.2	48.5	100

### Statistical analyses

SAS 9.0 (SAS Institute, Inc., Cary, NC, USA) was used for the statistical analyses, and all data are expressed as mean ± SD. One-way analysis of variance with the multiple range test was used to compare the difference in parameters among the groups, where *P* < 0.05 or *P* < 0.01 represented statistical significance.

## Results

### Clinical signs and gross lesions following inoculation of FAdV-4 in ducks

**Figure 1:**
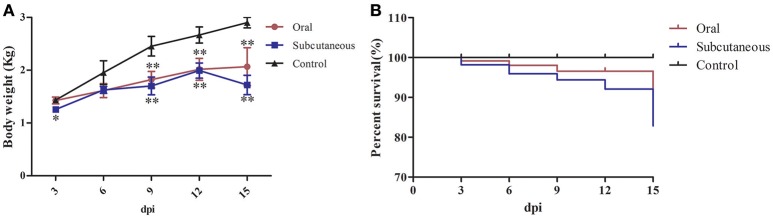
Body weight **(A)** and survival curve **(B)** of different groups. The data were processed by GraphPad Prism 5.0 (GraphPad Software Inc., San Diego, CA, USA). The comparison was between infected groups (i.e., oral or subcutaneous group) and the control group at the same day post-infection (dpi). ^*^Indicates that the difference between the infected groups and control group was significant (*P* < 0.05). ^**^Indicates that the difference between the infected groups and control group was extremely significant (*P* < 0.01). Significant differences were calculated by one-way analysis of variance using Duncan's multiple range test (SAS Institute, Inc., Cary, NC, USA).

None of the ducks in any group showed clinical symptoms at the early infection stage (i.e., 3 dpi); however, about 50% of the ducks in the oral group showed listlessness, stunted growth, and reduced appetite at 6 dpi, and one dead duck was found at 3, 6, and 9 dpi, respectively, and two at 15 dpi. In the subcutaneous group, most of the ducks showed listlessness, fluffed feathers, dropped wings, and stunted growth at 6 dpi; one dead duck was found at 6, 9, and 12 dpi, and two at 3 and 15 dpi. No dead ducks were found in the control group. Figure 1 provides the body weight and survival status of the above groups.

**Figure 2:**
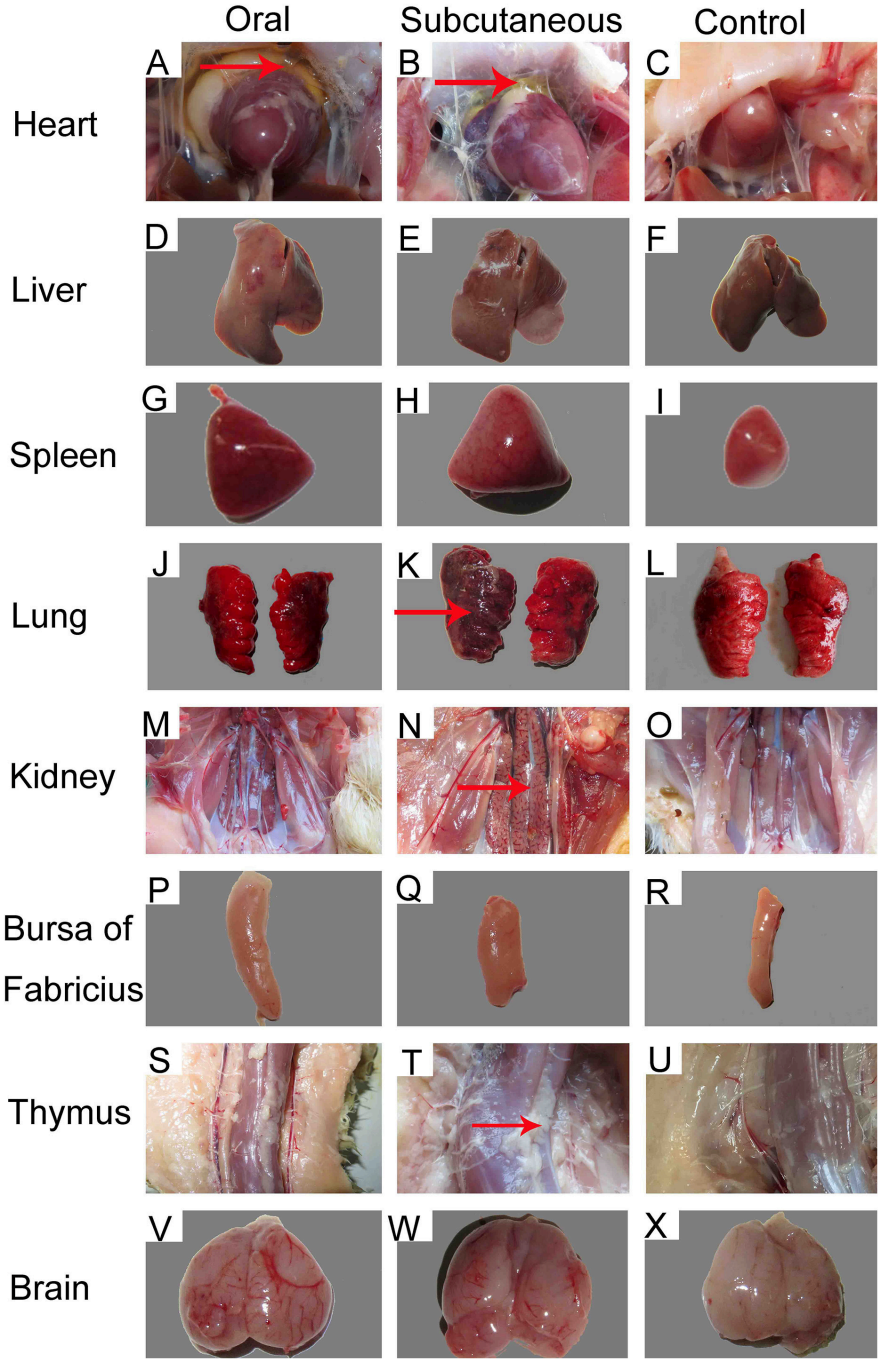
Gross lesions and postmortem changes in ducks infected with FAdV-4 (12 days post-infection [dpi]). **(A,B)** severe hydropericardium accompanied by heart yellow coronary fat; **(D,E)** enlarged, crisp livers with a pale yellow appearance; **(G,H)** swelling and spotted bleeding in spleen; **(J, K, M, N)** edema with severe congestion in lungs and kidney; **(P,Q)** swelling in bursa of Fabricius; **(S,T)** proliferation in thymus; **(V,W)** edema with hemorrhage in brain. No significant clinical signs or gross lesions were found in the control **(C, F, I, L, O, R, U, X)**.

Importantly, at 12 dpi, postmortem examination of the infected ducks showed severe pericardial effusion accompanied by heart yellow coronary fat (Figures 2A,B) and enlarged, edematous livers with bleeding spots (Figures 2D,E). In addition to the above typical clinical symptoms, the FAdV-4-infected ducks also presented significant lesions characterized by swollen and bleeding spots in spleen (Figures 2G,H), edema with varying degrees of congestion in the lungs and kidney (Figures 2J,K,M,N), swelling in the bursa (Figures 2P,Q), abnormal hyperplastic in the thymus (Figures 2S,T), and edema with hemorrhage in the brain (Figures 2V,W). No significant clinical signs or gross lesions were found in the control group (Figures 2C,F,I,L,O,R,U,X).

### Histopathology observation

**Figure 3:**
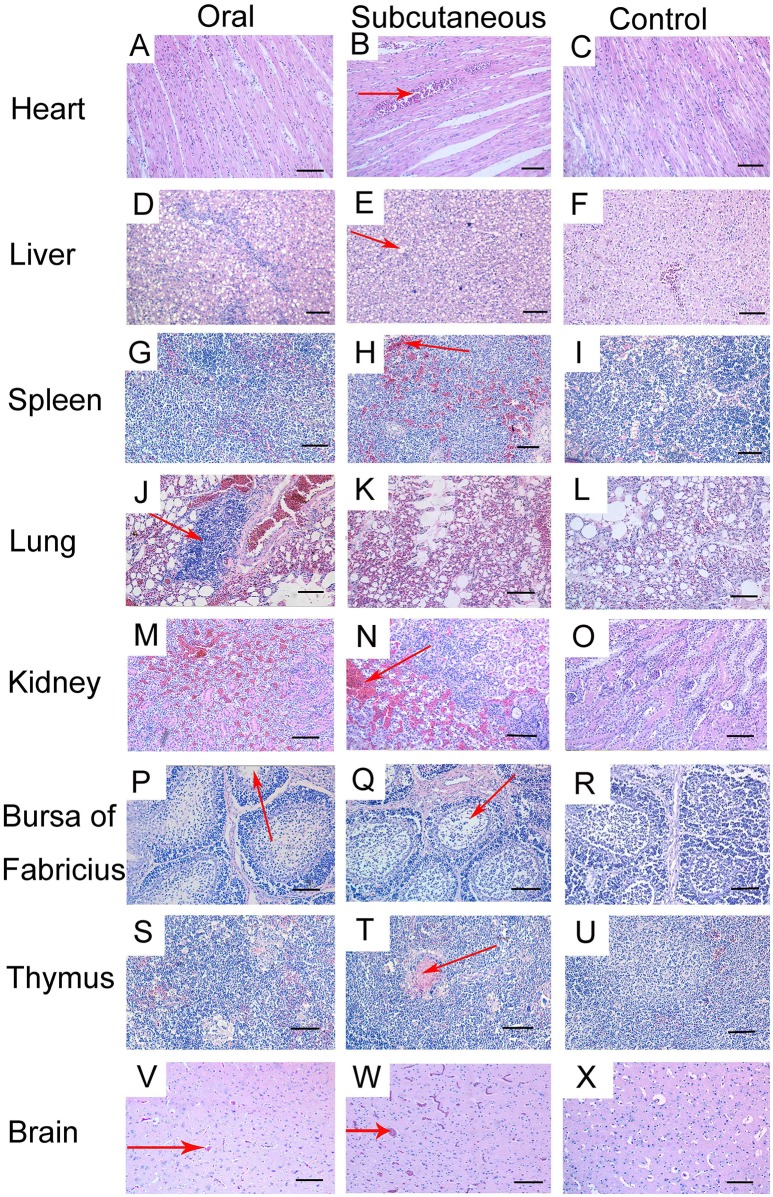
Histopathologic changes in viscera tissue of meat ducks infected with FAdV-4 (12 days post-infection [dpi]). **(A,B)** heart, slight granular degeneration, congestion, and dilated intercellular space; **(D,E)** liver, obvious fatty degeneration and basophilic inclusion bodies presented in hepatocytes, tissue structural disorders; **(G,H)** spleen, various degrees of hemorrhage; **(J,K)** lung, blood capillary congestion of pulmonary alveoli, multifocal areas with lymphocytes gathering; **(M,N)** kidney, enlargement and degeneration of glomerulus, narrowed glomerular sac, severe congestion accompanied by excessive lymphocyte infiltration; **(P,Q)** bursa, lymphocyte reduction and vacuole formation; **(S,T)** thymus, lymphocyte depletion; **(V,W)** brain, hemorrhage and edema. No pathological changes were found in the control group **(C, F, I, L, O, R, U, X)**. Scale bar = 100 μm.

Histopathological changes were detected in the heart, liver, lung, kidney, bursa, and brain of the FAdV-4-infected ducks. At 12 dpi, slight granular degeneration, congestion, and dilated intercellular spaces were showed in the heart (Figures 3A,B). Obvious fatty degeneration and basophilic inclusion bodies were presented in the hepatocytes, and structural disorders were discovered in the liver (Figures 3D,E). Severe hemorrhage, lymphocytes reduction and necrosis showed in the spleen (Figures 3G,H). Blood capillary congestion in the pulmonary alveoli, and multifocal areas containing lymphocytes in the lung (Figures 3J,K). In the kidney, the glomerulus was enlarged, the glomerular sac had narrowed, and there was severe congestion accompanied by excessive lymphocyte infiltration (Figures 3M,N). Lymphocytes were reduced and vacuoles were formed in the bursa and thymus (Figures 3P,Q,S,T). Besides, the brain exhibited hemorrhage and edema (Figures 3V,W). No pathological changes were found in the control group (Figures 3C,F,I,L,O,R,U,X).

### FAdV-4 antibody

**Figure 4:**
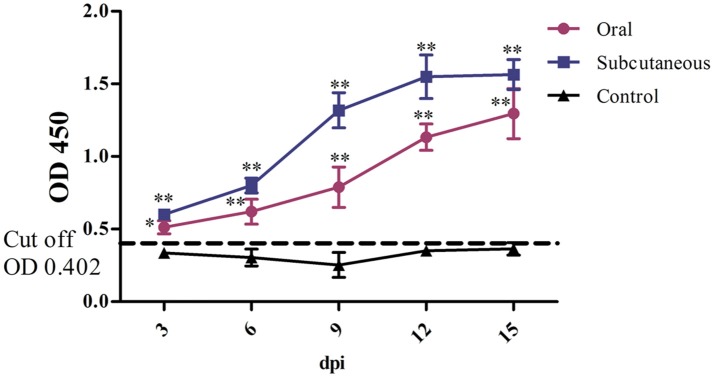
FAdV-4 antibody levels in the different groups. The comparison was between the infected groups (i.e., oral or subcutaneous group) and the control group at the same number of days post-infection (dpi). ^*^P <0.05, ^**^P <0.01. OD, optical density. The same as below.

FAdV-4 antibody levels in the duck serum were detected throughout the experiment. As Figure 4 shows, the antibody level in the oral group continued to increase throughout the experiment; however, the antibody level in the subcutaneous group peaked at 12 dpi and was maintained till 15 dpi. Notably, the serum FAdV-4 antibody levels were higher in the infected groups than in the control group (*P* < 0.05 or *P* < 0.01) at 3–15 dpi.

### IL-2 and IFN-γ

**Figure 5:**
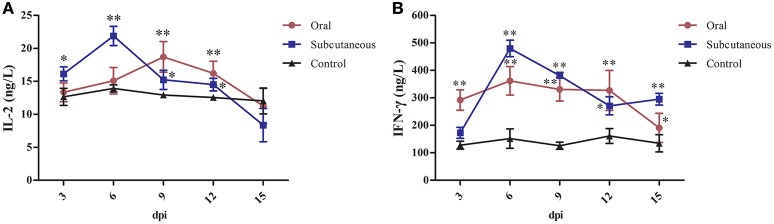
Interleukin (IL)-2 **(A)** and interferon (IFN)-γ **(B)** levels in different groups. (^*^P <0.05, ^**^P <0.01).

As Figure 5 shows, IL-2 levels in the subcutaneous group peaked rapidly at 6 dpi but began to decrease at 15 dpi; these levels were significantly higher than in the control group (*P* < 0.05 or *P* < 0.01, respectively) at 3–12 dpi; however, the IL-2 levels in the oral group increased slowly and peaked at 9 dpi and were significantly higher than in the control group (*P* < 0.01) at 9 and 12 dpi.

IFN-γ levels in the subcutaneous group peaked rapidly at 6 dpi and then began to decrease at 12 dpi; these levels were significantly higher than in the control group (*P* < 0.01) at 6–15 dpi; however, IFN-γ levels in the oral group changed only slightly and tended to decline at 15 dpi, although the IFN-γ levels in this group were higher than in the control group (*P* < 0.05 or *P* < 0.01) at 3–15 dpi.

### ALP, ALT, AST, LDH, and urea levels

**Figure 6:**
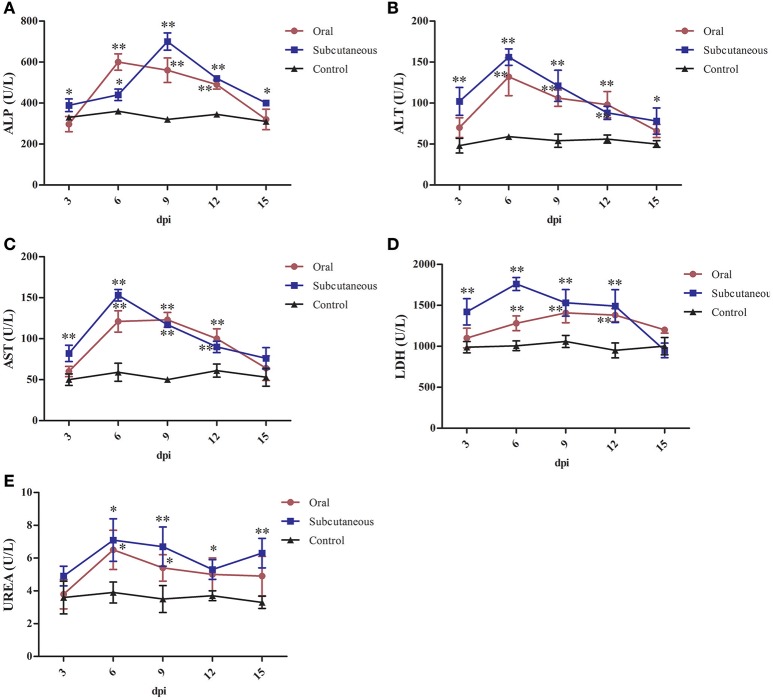
Alkaline phosphatase (ALP) **(A)**, alanine transaminase (ALT) **(B)**, aspartate transaminase (AST) **(C)**, lactate dehydrogenase (LDH) **(D)** and urea **(E)** levels in different groups. (^*^P < 0.05, ^**^P < 0.01).

As Figure 6 shows, the serum ALP, ALT, AST, and urea levels in the oral group peaked at 6 dpi and the LDH levels peaked at 9 dpi. All these levels were significantly higher than in the control group (*P* < 0.05 or *P* < 0.01) at 6–12 dpi.

The serum ALT, AST, LDH, and urea levels in the subcutaneous group peaked at 6 dpi and the ALP levels peaked at 9 dpi. All these levels were significantly higher than in the control group (*P* < 0.05 or *P* < 0.01) at 3–12 dpi.

### Virus DNA concentration

**Figure 7:**
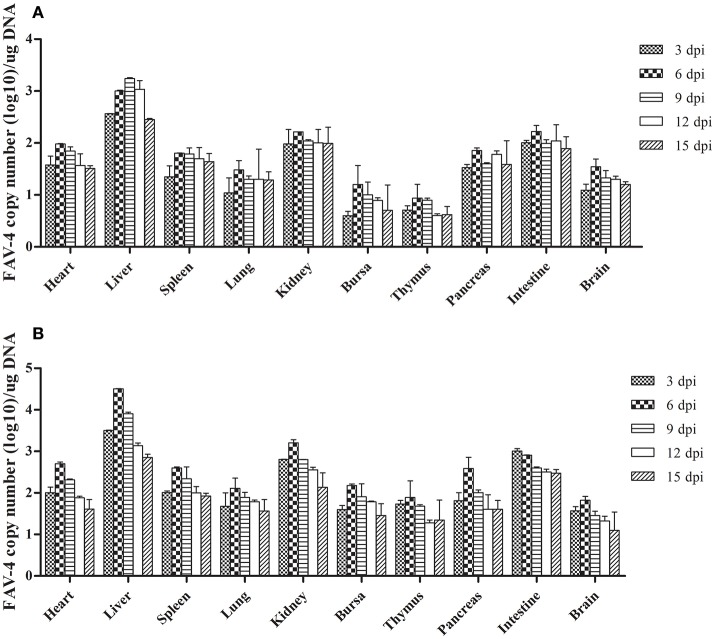
FAdV-4 viral DNA concentration in visceral tissues samples at different dpi (**A**: oral group; **B**: subcutaneous group).

As Figure 7 shows, the number of FAdV-4 copies peaked at 6 dpi in all the tested tissues from infected ducks, except in the livers of the oral group and the intestines of the subcutaneous group. The number of copies in most of the tested tissues began to decline at 9 dpi. Notably, more FAdV-4 copies were found in the liver, kidney, and intestine than in other tissues from the oral and subcutaneous groups. Moreover, when these two groups were compared, the overall number of virus copies in the tested tissues was higher in the subcutaneous group than in the oral group. No virus DNA was detected in the control group.

### Sequence comparison of hexon gene

As Table 2 shows, the hexon genome of the SDSX strain shared 53.9–99.9% of the nucleotide and 47.2–99.9% of the amino acid sequences with that of representative strains from different lineages.

**Figure 8:**
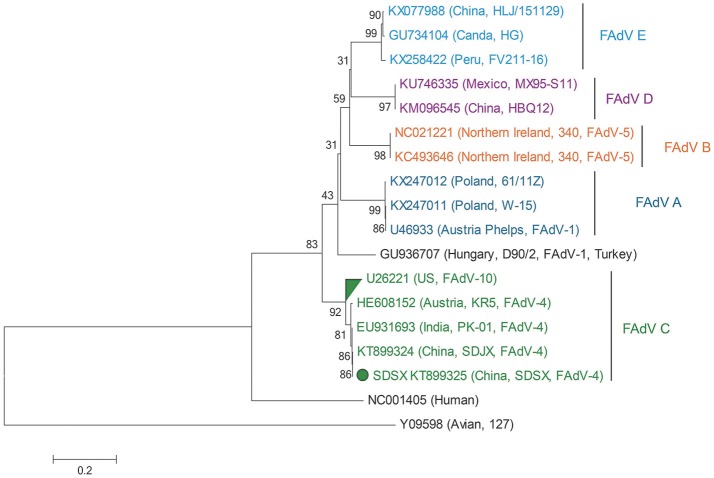
Phylogenetic tree comparing the hexon gene nucleotide sequence of the SDSX isolate with that of other avian strains. The sequence corresponding to the virus isolated at SDSX is marked by a green dot. The phylogenetic tree was built using neighbor-joining analysis and MEGA 6.0, and the bootstrap confidence values were determined using 1,000-bp replicates.

Notably, the SDSX strain shared 99.8% of the nucleotide and amino acid sequences with the India-isolated strain (EU931693, PK-01), which indicated that this strain might have originated from early India isolates (Table 2, Figure 8).

## Discussion

FAdV-4, one of the 12 FAdV serotypes, is the pathogenic agent of HHS, which is an acute infectious disease in fowl characterized by HHS, IBH, spleen and kidney enlargement, and hemorrhagic lungs, and is fatal in 5–20% of the infected birds. In recent years, HHS outbreaks caused by FAdV-4 have occurred in several countries, resulting in huge economic losses or hazards for the poultry industry (Hafez, 2011; Kaján et al., 2013; Kataria et al., 2013; Niczyporuk, 2016); however, few studies have reported on the pathogenicity of FAdV-4 in meat ducks, except one on an intracerebral infection that caused 15% mortality (Chen et al., 2016) and another on an oral infection that showed no clinical signs (Pan et al., 2017). Therefore, the pathogenesis of the virus in ducks is not clearly understood, and it is necessary to better understand the pathogenic mechanisms of FAdV-4 in meat ducks to effectively control a viral epidemic.

In this study, we chose 25-day-old meat ducks as the experimental animal for evaluating HHS because the disease usually occurs at this approximate age in the surviving ducks of diseased flocks (Chen et al., 2016). Regardless of the inoculation method (i.e., oral or subcutaneous), the typical symptoms of HHS were easily observed in the infected groups, indicating that we established an animal regression experiment for FAdV-4 successfully.

From the clinical symptoms, gross lesions, body weight, and percent survival, we observed that ducks in the subcutaneous group were more impacted than those in the oral group. This result may be because subcutaneous virus inoculation has been reported to be more effective than oral virus inoculation (Yu et al., 2017). In the subcutaneous group, the virus particles directly entered the peripheral blood and reached all tissue organs through the blood within a short period of time, resulting in relatively serious damage; however, in the oral group, the virulence of the virus was decreased because of various digestive enzymes in the digestive system; this might have impeded virus colonization, resulting in less damage to the organism.

We detected virus DNA in all the tested tissues at an early infection stage (3 dpi; Figure 7), implying that the virus could quickly invade and replicate in many tissues, including the brain. The liver contained the highest concentration of FAdV-4, indicating that it might be the main target organ of this virus, which was consistent with the other latest reports (Pan et al., 2017). Hence, liver cells might also be the prime organ for the proliferation of FAdV-4.

Furthermore, several studies have documented that virulent FAdV-4 strains have a predilection for lymphoid tissues, resulting in the depletion of lymphocytes in lymphoid organs (Schonewille et al., 2008). This might be the reason for vacuolation in the bursa of infected ducks (Figures 3N,R). Notably, the virus DNA was detected in the brains of infected ducks at 3 dpi (Figure 7), indicating that FAdV-4 could easily pass through the blood–brain barrier. Rapid and high levels of virus invasion of the tissue might be the reason for death in some infected ducks; however, the infected ducks did not show any symptoms of nerve damage, the reasons for which require further research.

IL-2, also named T-cell growth factor, is the primary cytokine for regulating the body's anti-inflammatory effects in cellular immunity (Abdul-careem et al., 2006; Bayer et al., 2013), the IL-2 concentration will decrease when the body is in an immunosuppressive stage (Yu et al., 2018). In addition, IFN-γ, produced mainly by thymus-dependent lymphocytes and natural killer cells, regulates the body's antivirus responses through cellular immunity processes (Hu et al., 2016). In this study, to evaluate the effects of this virus on ducks' immunity, we chose the FAdV-4 antibody as a reliable indicator for humoral immunity and used IL-2 and IFN-γ as representative indices of cellular immunity. Notably, FAdV-4 antibody levels in the oral group increased at the early infection stage, peaked at 12 dpi, and were maintained for a long duration, which was different from the results of Pan et al., suggesting that the FAdV-4 antibody titers in orally infected ducks peaked at 14 dpi and then sharply decreased. These different results might be attributed to the difference in ages of the ducks during infection (i.e., 35 days old in Pan et al.'s study). In this study, the long duration and high levels of FAdV-4 antibody titers indicated that humoral immunity plays an important role in host antiviral properties.

In addition, we found that after the initial increase, IL-2 and IFN-γ levels decreased over time (Figure 5), which could have been related to the fact that the ducks were in a normal immune state at the beginning of inoculation; however, the immune organs of the infected ducks became seriously damaged with the constant invasion and colonization of the SDSX strain and resulted in the decrease in IL-2 and IFN-γ levels. Taken together, this FAdV-4 strain severely impacted the humoral and cellular immunities of the infected ducks, which resulted in immunosuppression (Schonewille et al., 2008).

Serum ALP, one of the indicators of cirrhosis, is mainly produced by the liver, hepatocytes will overproduce ALP if the hepatobiliary function is abnormal (Witters et al., 2008; Yu et al., 2018). ALT mainly exists in the hepatocytes, and only 1% of liver cell necrosis leads to a doubling of ALT levels in serum; therefore, ALT is often used as the most sensitive indicator of liver function (Kew, 2000; Guo et al., 2013). When hepatocytes and myocardial cells are damaged, AST levels increase rapidly (Qiu et al., 2009; Zhu et al., 2009). LDH exists in the cytoplasm of all histiocytes in body, and the levels of it mainly reflect tissue damages, which increases as long as the myocardial muscle, skeletal muscle, kidney, and liver are injured (Giannini et al., 2005; Yu et al., 2018). Urea is a key indicator of renal function. An increase in urea levels indicates renal dysfunction (D'Apolito et al., 2015).

In this study, the levels of ALP, ALT, AST, LDH, and urea in the serum of the infected groups were generally higher than those in the control group and showed a trend of first increasing and then decreasing after a period of time. This might be the result of liver cell damage caused by the virus, such as severe fatty degeneration and disorders of structural tissue (Figures 3B,F); this causes release of various enzymes in the cells into the blood. As the tissue cells continuously repair themselves, the enzyme levels in plasma decrease. This results were analogous to that of previous studies (Asrani et al., 1997; Guo et al., 2013); therefore, it was observed that FAdV-4 infection damages the functions of the liver, kidney, and heart to a certain degree.

Hexon, one of the main structural/antigenic proteins of FAdV-4, has multiple antigenic determinants of genotype, species, and subspecies, and is often used to analyze the genetic evolutionary relationship of FAdV-4 (Luo et al., 2012)(Marek et al., 2010; Chen et al., 2016; Pan et al., 2017). According to our nucleotide sequence analysis of the hexon gene of the SDSX strain and of 16 other representative strains of different lineages, the SDSX strain shared nearly 100.0% nucleotide and amino acid sequence identity with the India-isolated strain (EU931693, PK-01; Table 2, Figure 8); therefore, we suggest that this strain could have originated from the earlier India isolates.

In conclusion, FAdV-4 (SDSX) induces the upregulation of antibody titers, IL-2, and IFN-γ expression in 25-day-old meat ducks at the early infection stage and causes damage to the tissues of many immune organs, especially the liver and kidney. In addition, the above data provide useful information for further studies on the pathogenicity of FAdV and contribute to the control of the disease epidemic.

## Author contributions

GY, YW, MZ, and YL completed most of the experiments. YT and YD designed experiments and reviewed the manuscript.

### Conflict of interest statement

The authors declare that the research was conducted in the absence of any commercial or financial relationships that could be construed as a potential conflict of interest.
